# *Varroa destructor* mites vector and transmit pathogenic honey bee viruses acquired from an artificial diet

**DOI:** 10.1371/journal.pone.0242688

**Published:** 2020-11-24

**Authors:** Francisco Posada-Florez, Eugene V. Ryabov, Matthew C. Heerman, Yanping Chen, Jay D. Evans, Daniel E. Sonenshine, Steven C. Cook

**Affiliations:** 1 Bee Research Laboratory, Beltsville Agricultural Research Center, United States Department of Agriculture—Agricultural Research Service, Beltsville, Maryland, United States of America; 2 Department of Biological Sciences, Old Dominion University, Norfolk, Virginia, United States of America; University of North Carolina at Greensboro, UNITED STATES

## Abstract

The ectoparasitic mite *Varroa destructor* is one of the most destructive pests of the honey bee (*Apis mellifera*) and the primary biotic cause of colony collapse in many regions of the world. These mites inflict physical injury on their honey bee hosts from feeding on host hemolymph and fat body cells/cellular components, and serve as the vector for deadly honey bee viruses, including Deformed wing virus (DWV) and the related Varroa destructor virus-1 (VDV-1) (*i*.*e*., DWV-like viruses). Studies focused on elucidating the dynamics of *Varroa*-mediated vectoring and transmission of DWV-like viruses may be confounded by viruses present in ingested host tissues or the mites themselves. Here we describe a system that includes an artificial diet free of insect tissue-derived components for maintaining *Varroa* mites for *in vitro* experimentation. Using this system, together with the novel engineered cDNA clone-derived genetically tagged VDV-1 and wild-type DWV, we demonstrated for the first time that *Varroa* mites provided an artificial diet supplemented with engineered viruses for 36 hours could acquire and transmit sufficient numbers of virus particles to establish an infection in virus-naïve hosts. While the *in vitro* system described herein provides for only up to five days of mite survival, precluding study of the long-term impacts of viruses on mite health, the system allows for extensive insights into the dynamics of *Varroa*-mediated vectoring and transmission of honey bee viruses.

## Introduction

The ectoparasitic mite, *Varroa destructor* (Anderson and *Trueman*, *2000*) (Acari: *Varroidae*), is the most harmful pest of the European honey bee, *Apis mellifera* (Linnaeus, 1758) (Insecta: *Apidae*). Importantly, *Varroa* mites vector a number of honey bee viruses [1], including DWV-A [2], *Varroa destructor* virus-1 (VDV-1, or DWV-B) [3] and DWV-VDV-1 recombinants [4–7] (*i*.*e*., DWV-like viruses). These viruses, together with *Varroa* mites, are the most prevalent and widespread honey bee pathogens and linked to declines in honey bee health and populations worldwide [1, 8–10]. DWV-like viruses are transmitted both vertically [11] and horizontally [12], the latter of which includes the special case of *Varroa*-mediated transmission. *Varroa*-mediated transmission of DWV-like viruses can affect the genetic diversity and elevate the virulence of DWV-like virus populations in honey bees [6, 12, 13], thus increasing the potential harm they inflict to colonies. Despite their obvious importance, the mechanisms that underlie *Varroa*-mediated vectoring and transmission of DWV-like viruses to honey bees remain poorly understood. However, it is known that strains of viruses can differ in their ability to replicate in *Varroa* mites; while DWV-A appears to not replicate during vectoring by *Varroa* mites [14], VDV-1 appears to do so [15].

Detailed studies of the vectoring and transmission of DWV-like viruses to honey bees can be complicated by the interactions among multiple strains and recombinants comprising virus populations. Additionally, nearly all *Varroa* mites have ‘background’ levels of potentially non-biologically active DWV-like viruses, which can be high, and may mislead results from molecular studies of *Varroa*-mediated vectoring and transmission of DWV-like viruses [14]. Robust systems for tracking the growth and transmission routes for specific viruses as single strains, recombinants, or mixed infections, are urgently needed.

Successful research into the dynamics of *Varroa*-mediated vectoring and transmission of DVW-like viruses to honey bees requires: 1) specific viral strains that are genetically tagged that allow tracking of the viruses during vectoring and transmission, and 2) an *in vit*ro system for maintaining *Varroa* mites that includes an artificial diet that is free of honey bee cells and cellular components possibly having virus aggregates. Recently developed genetically tagged DWV-like viruses can be used to distinguish between *Varroa-*transmitted tagged and inevitable ‘background’ wild-type viruses and for tracking specific strains and/or recombinants of DWV-like viruses [13–17]. A system that maintains *Varroa* mites by feeding them an artificial liquid diet via a membrane has been the subject of investigation over several decades [18, 19]. Many previous attempts to maintain mites *in vitro* employed stretched-thin (~10 μm) parafilm [18, 20] or synthesized chitosan [19] membranes, through which the mites’ short mouthparts [21] would access a liquid diet. The devices used varied, but some were made of specialized materials [18]. Although with some systems *Varroa* mites survived for up to five days, and some also laid eggs, leakage and/or microbial contamination of diets reduced mite viability, thus precluding their use in experiments requiring larger sample size [19, 22].

In this report, we describe a system developed for maintaining *Varroa* mites for *in vitro* experimentation. The system is comprised of a device made of simple, common laboratory components, a parafilm feeding membrane, and a diet having no components derived from honey bees or any other insects tissues that may contain viruses. This system is particularly suitable for research of *Varroa*-mediated vectoring and transmission of DWV-like viruses. We used this system and a novel genetically tagged cDNA clone for *Varroa destructor virus-1* (VDV-1 or DWV-B) to address a key question in honey bee-virus interactions. Specifically, we tested whether VDV-1 particles introduced to the diet could persist and be acquired by the feeding mites, then subsequently transmitted to pupae leading to an established infection.

## Materials and methods

### Design of infectious cDNA clone of *Varroa destructor* virus-1 (VDV-1) and production of clone-derived inoculum

We designed a full-length infectious cDNA clone of a Californian VDV-1 isolate, GenBank Accession number MN249174 (S1 Text). The cDNA had an introduced genetic marker, an *Asi*SI restriction site, at position 277 nt, which distinguished the clone-derived VDV-1 from wild-type VDV-1 strains, thereby allowing us to trace transmission of this VDV-1 isolate. The VDV-1 cDNA clone and clone-derived infectious virus particles were produced using a previously described approach [13] and detailed in S1 Text. In brief, the full-length cDNA clone of the virus was produced using total RNA from honey bees sourced from California in 2016 (isolate CA-07-2016), which showed high VDV-1 and low DWV levels [23]. Both the two overlapping cDNA fragments amplified by RT-PCR using specific primers and the synthetic gene corresponding to the 277 nt 5’ part of the genomic RNA were assembled together in a plasmid vector (S1 Table; S1 Text). The resulting full-length VDV-1 cDNA plasmid construct was used to prepare the template for in *vitro* transcription. To produce clone-derived VDV-1 inoculum, the purified *in vitro* RNA full-length VDV-1 transcript generated using the linearized VDV-1 cDNA plasmid was injected into the hemolymph of purple-eyed stage honey bee pupae. Pupae were incubated for 4 d at +33 ^o^C with 82.2 ±1.3% (SE) relative humidity (RH) to allow propagation of the clone-derived virus infection. Tissue extracts containing the clone-derived VDV-1 virus particles were collected by homogenizing infected pupae in PBS, and then filtering the supernatant through a 0.22μm nylon syringe filter (Thermo Fisher, Waltham, MA). The VDV-1 extract introduced to the artificial diet (below) contained the clone-derived VDV-1 (1.7 × 10^8^ per μL) and background wild-type DWV (1.6 × 10^5^ per μL) derived from the recipient pupae (see, S1 Text for additional details).

### Formulation of the V-BRL diet

The base composition of the artificial diet, hereafter termed the V-BRL (Varroa-Bee Research Lab) diet was similar to that reported by [19] and [20]. Briefly, the diet included 30% Schneider’s medium, 30% CMRL-1000, 1% Hanks salt solution (without NaCO_3,_ CaCl or MgSO_4_), 10% bovine serum, 4% Insect medium supplement (cell culture type), 10% TC-100 Insect medium (with glutamine and NaCO_3_) and 15% sterile water (all from Sigma-Aldrich, St. Louis MO). To minimize contamination, fresh diet was prepared in a sterile hood and stored frozen (-20 ^o^C) in small aliquots. No antibiotics or antifungal agents were included to avoid compromising microbiota that may provide essential nutrients for mite nutrition since species of *Diplorickettsia*, *Arsenophonus*, *Morganella*, *Spiroplasma*, *Enterococcus*, and *Pseudomonas* all have been reported to inhabit *Varroa* mites [24].

### Designs of device and feeding membrane sachet

**Fig 1:**
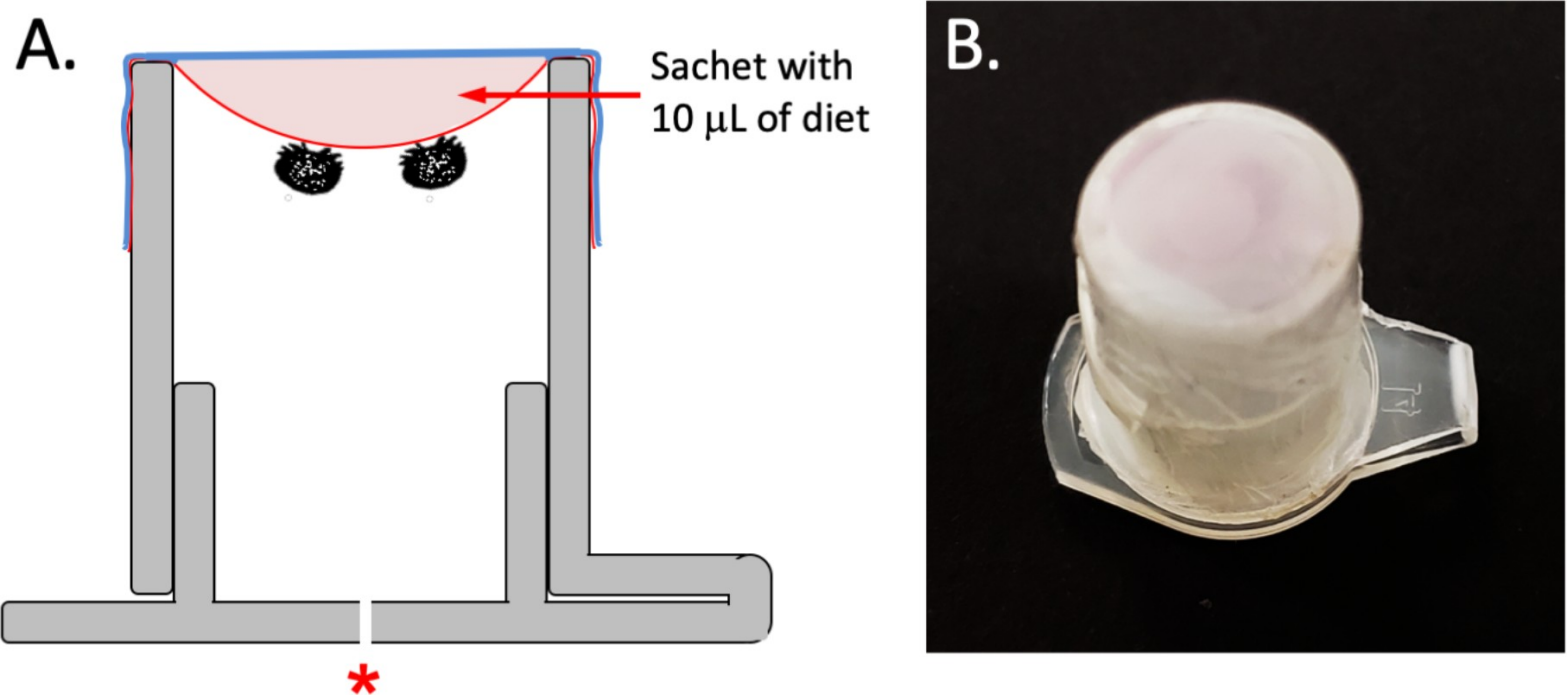
Details of the device and membrane sachet comprising the in-vitro system used for maintaining *Varroa* mites during experiments. (A). Diagram showing the design of the device housing the mites, as modified from a snap-cap 1.5 mL microcentrifuge tube. The arrow shows parafilm membrane sachet with diet. A red asterisk indicates a hole in the lid for air pressure relief. Two mites are shown attached to the inner membrane to feed on the diet. (B) Photo of the complete system, comprising the device housing the mites and attached parafilm membrane sachet filled with 10 μL of artificial diet solution.

Snap-cap polypropylene 1.5 mL microcentrifuge tubes were chosen for constructing the device housing the mites (Fig 1). The tubes were cut crosswise near the mid-point that allowed a 1 cm diameter opening. Cut surfaces were smoothed with sandpaper to avoid damaging the parafilm (American National Can Company, NY) membranes that would later be attached to the cut edge of the tubes. The devices were sterilized prior to attaching the feeding membrane sachet; while wearing sterile nitrile gloves, devices were transferred to a sterile laminar flow hood, disinfected for 2 min with 1.0% bleach, followed directly by 2 min in 70% ethanol and 2 min rinsing in sterile water, and then exposed for 1 h to ultraviolet light. Following disinfection and while still in the hood, each device was covered with a thin sheet of sterilized parafilm stretched to an average thickness of 16.6 ±5.86 μm and wrapped over the open end of the device. The membrane thickness was checked with a Marathon digital micrometer (Marathon Corp., Canada). Ten microliters of the diet solution were pipetted onto the center of the parafilm membrane, and to complete the sachet, the second layer of sterile parafilm was sealed over the membrane plus diet solution [25–27]. The snap-cap lid on the other side of the device was opened to insert a female *Varroa* mite. Before closing, the lid was punctured with a sterile no. 1 stainless steel pin to form a minute hole (approximately 0.5 mm) to allow air to escape so the parafilm sachet would not rupture when the lid was snapped shut (Fig 1). The completed devices were placed in a dark incubator (Thermofisher Scientific, San Jose, CA) set at 32.1 ± 0.3°C, and having 82.2 ± 1.3% RH.

### Obtaining *Varroa* mites and honey bee pupae for experiments

All mites and honey bee pupae used in these experiments were obtained from honey bee colonies from apiaries on the grounds of the USDA-ARS Beltsville Agricultural Research Center (BARC; Beltsville, Maryland, USA). The sugar roll method [28] was used to collect phoretic mites from honey bee colonies that had not been subject to miticidal treatments. Following collection, and after rinsing mites with tap water_,_ they were placed on Petri dishes lined with dry tissue paper. Mites were checked for activity prior to experimental selection, and only active mites were used. Honey bee pupae were obtained from frames of capped worker brood sourced from honey bee colonies regularly treated with miticides (Apivar®, Mann-Lake, USA) according to manufacture instructions. White-to-red eyed stage pupae were collected by first using a pin tool to remove the wax cap, then using soft forceps (BioQuip, USA), pupae were grasped by the thorax and gingerly lifted out of their cells. Only *Varroa* mite-free pupae were selected, and were used immediately for experiments then kept in an incubator (as above).

### Testing device performance and *Varroa* mite longevity

Three trials were conducted to evaluate the suitability of the system for maintaining mites for experimentation. In the first trial, ten replicates were prepared with three *Varroa* mites in each device. In the second trial, ten replicates were prepared; six with three mites and four with four mites, for a total of 34 mites. In the third trial, 15 replicates were prepared; four devices with two mites, four with three mites and seven with four mites each for a total of 42 mites. In each trial 10μL of diet containing fluorescent microbeads were placed within the sachet. As a positive control for survival, female *Varroa* mites were allowed to feed on honey bee pupae. Pupae were collected from beehives, placed in clear gelatin capsules (Capsuline, Pompano Beach, FL) with several mites, and mite and bee survival were monitored daily. Pupae were replaced after 3–4 d with fresh bee pupae to ensure that the pupal hosts had not deteriorated, transformed into an adult bee, or were otherwise unsuitable, as described previously [29]. As a negative control for mite survival, mites were housed in the devices with membrane sachets containing only water. Replicate devices and controls were incubated as described above. Mite mortality was recorded daily. Mites were recorded as ‘dead’ when they showed no physical response to gentle prodding by a fine paint brush. Also, daily visual inspections of devices were made for obvious signs of microbial contamination, excessive diet removal by leakage or evaporation of the diet, and for obstructions blocking mites from accessing the diet.

### Confirming mites fed on the artificial diet

Because mites defecate frequently when allowed to feed [30], as a gauge of mite feeding, the number of mite excretory droplets deposits were recorded daily by inspecting the parafilm membrane and walls of the devices under a Zeiss Axioskop 2 Plus compound microscope (Zeiss Corp., Dublin, CA). Further confirmation of feeding came from examining mite feces collected from devices housing mites fed on artificial diets with and without the addition of fluorescent microbeads under a Zeiss Axio Imager.M2 (Dublin, CA) microscope set at 488 excitation/520 emission [30].

### Acquisition of viruses by *Varroa* mites from the artificial diet

**Fig 2:**
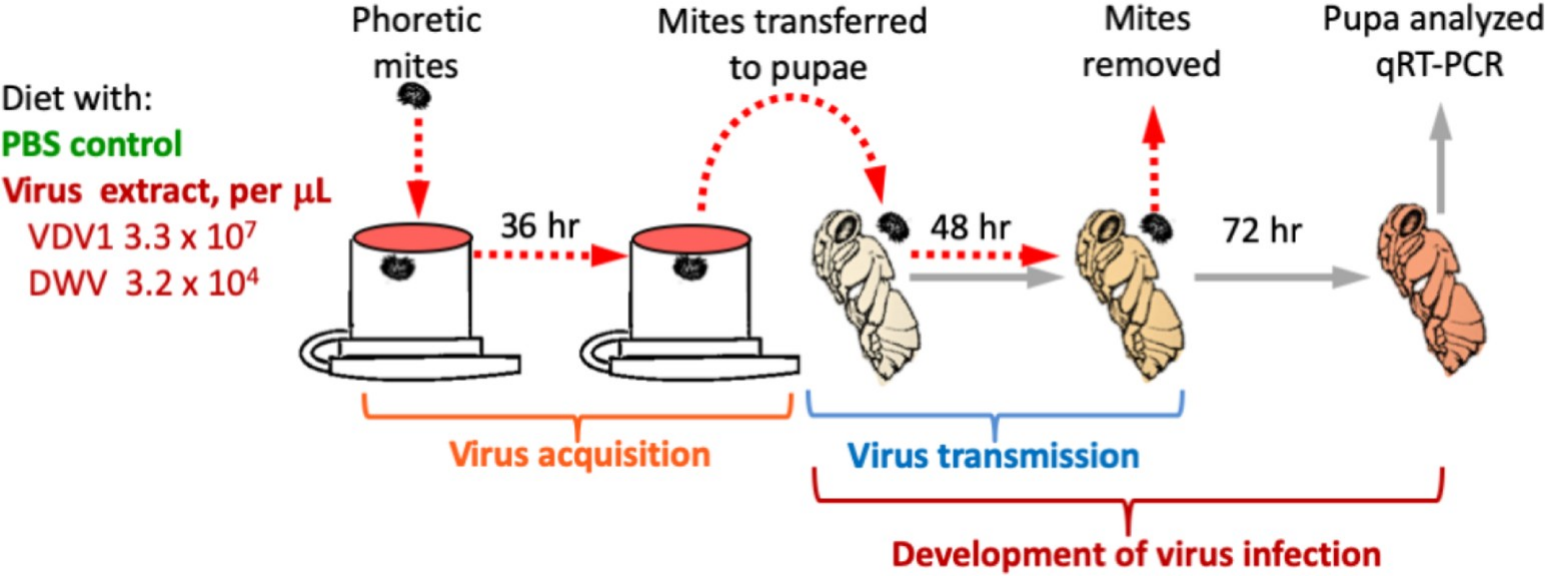
Application of the developed system for studying the vectoring of honey bee viruses by *Varroa* mites. A schematic representation of the experimental design is shown. Mites were allowed to feed for 36 h either on diet containing 1) cDNA clone derived particles of VDV-1, 3.3 x 10^7^genome equivalents per μL, 2) wild-type DWV, 3.2 x 10^4 ^genome equivalents per μL, or 3) a control diet containing PBS free of viral particles.

The filtered tissue extract introduced to the *Varroa* diet contained virus particles of the clone-derived VDV-1 at a concentration of 1.7 × 10^8^ genome equivalents/μL and background wild-type DWV derived from the recipient pupae used for recovery of a clone-derived VDV-1 at a concentration of 1.6 × 10^5^ genome equivalents/μL. We supplemented the artificial diet with filtered PBS tissue extract containing particles of the cDNA clone-derived VDV-1 and wild-type DWV (80% diet, 20% filtered PBS tissue extract) (Fig 2). The virus-supplemented diet contained 3.3×10^7^ genome equivalents of the clone-derived VDV-1 and 3.2 × 10^4^ genome equivalents of wild-type DWV per μL diet. The diet supplemented with PBS (80% diet, 20% PBS) containing no tissue extract was used as a control.

### Transmission of the acquired viruses to honey bee pupae

Following the 36h acquisition period the surviving mites were placed individually on a pink-eyed honey bee pupa housed in gelatin capsules and placed in a dark incubator (see above) for 48h to allow for the transmission of the virus to the pupae. Pupae were then incubated for an additional 72 h to allow virus replication (Fig 2). To determine whether viral transmission occurred, total RNA was extracted from each of the pupae from the experimental (n = 15) and control (n = 9) treatment groups. Next, the copy numbers of VDV-1 and DWV genomic RNA in extracts were assessed with reverse transcription-quantitative polymerase chain reaction (RT-qPCR) as reported in [23] and using the primers given in S1 Table. The levels of honey bee actin quantified by the RT-qPCR were used to normalize the viral copy numbers across experimental groups. To further demonstrate that the VDV-1 detected in the pupae was both derived from the cDNA clone and acquired by the mites feeding on the artificial diet, the RT-PCR fragment corresponding to the 5’ terminal 1200 nt was digested with the *Asi*SI restriction enzyme (New England Biolabs). The AsiSI restriction site, which is not present in the wild-type VDV-1 sequence, was introduced to the VDV-1 cDNA clone at position 281. The digested and undigested PCR fragments were separated by electrophoresis in 1.2% agarose gel and visualized using ethidium bromide staining.

### Statistical analyses

We compared the survival of mites from the three trials fed on the artificial diet to that of mites from both the positive and negative control groups using a Kaplan-Meier test (JMP, version 12, *SAS*, Cary, NC). The qRT‐PCR data were analyzed using one‐way analysis of variance (ANOVA) to determine whether significant variation existed between the copy number of DWV, VDV-1, and honey bee actin mRNA in the pupae of different treatments. The number of fecal pellets deposited per live mite per day was analyzed using one-way ANOVA.

## Results

### System integrity and *Varroa* mite survival

**Fig 3:**
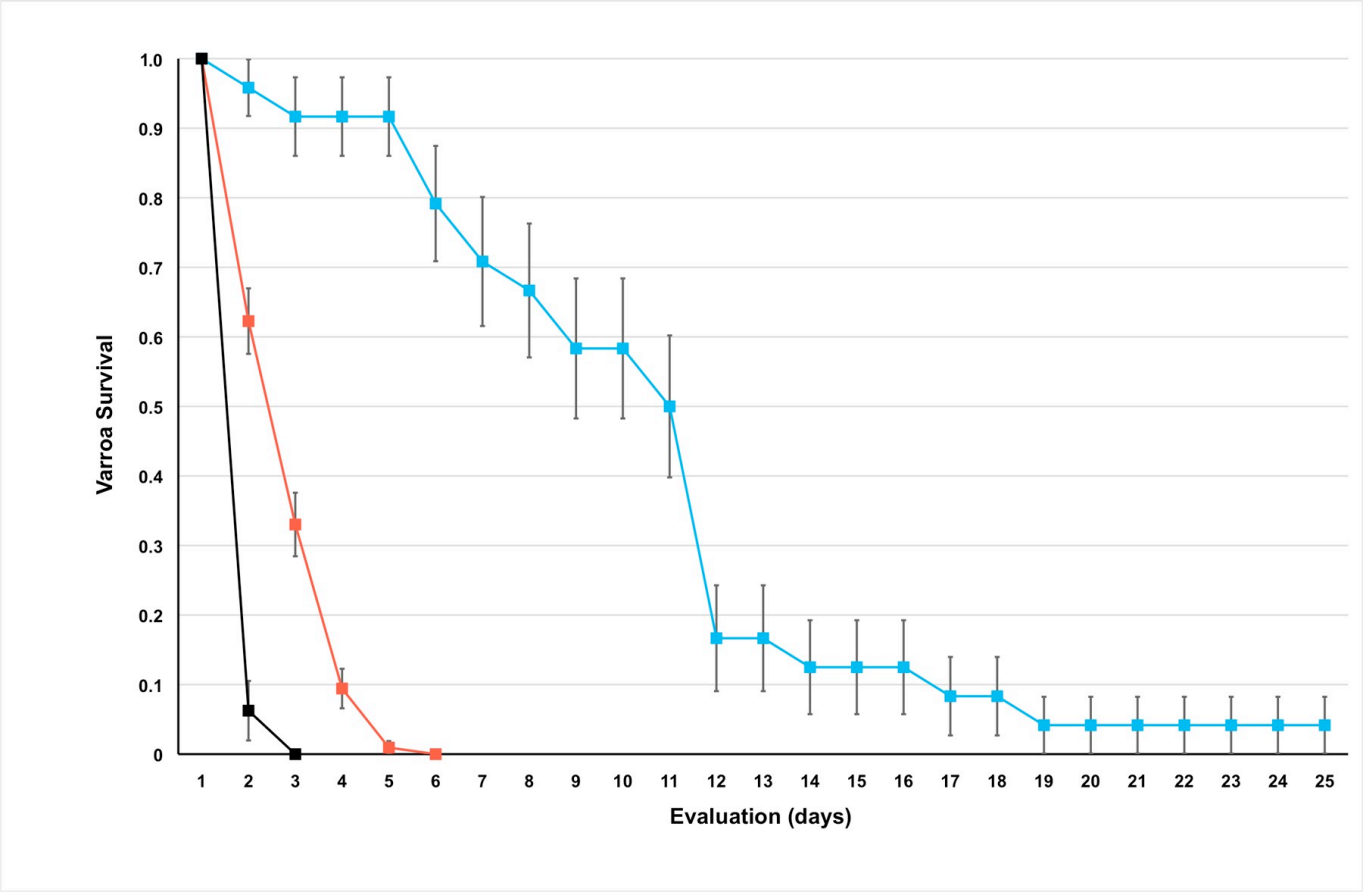
Trials testing the survival of *Varroa* mites fed on the artificial diet and positive and negative controls. Survival curves are shown for trials of *Varroa* mites fed the artificial diet (red line) and curves for mites feeding on honey bee pupae (blue line) and mites provided only water (black line). Data points on the graph represent the mean percent survival (± SE) in days for each trial.

The system (Fig 1) allowed *Varroa* mites to feed on the artificial diet without honey bee-derived components through a thin parafilm membrane. No evidence of microbial contamination, leakage or evaporation of the diet, or obstacles precluding mite’s access to the diet were observed. The mean survival times (± SE) for *Varroa* mites from the longevity trials are reported in Table 1; some mites survived 5 d feeding on the artificial diet (Fig 3; S2 Table).

**Table 1:** Survivorship data for mites from trials with artificial diet and positive and negative controls.

Treatment group	Number of mites	Mean survival time (days)	Standard error
Pupae (positive control)	24	9.42	0.90
Feeding device -Water(negative control)	32	1.06	0.04
Feeding device–Diet	106	2.10	0.09

Mean percent survival (± SE) for *Varroa* mites fed on honey bee pupae, artificial diet, or only water. The statistical significance of the results was determined by the Wilcoxon Chi-square test (*P* < 0.001, χ^2^ = 84.49).

In contrast to mites fed on the artificial diet, mites fed on bee pupae survived up to 25 days and had an average survival time of 9.42 days (Fig 3, blue line; S2 Table). Mites housed in the devices without diet survived an average of 1.06 ± 0.04 d and began dying 6 h of confinement; within 24 hours 32 of the 34 mites in the group had died, and the remaining two mites died by day two (Fig 3, black line). Contrary to poor survival by these mites, those fed on the artificial diet survived significantly longer (Table 1; Fig 3, red line).

**Fig 4:**
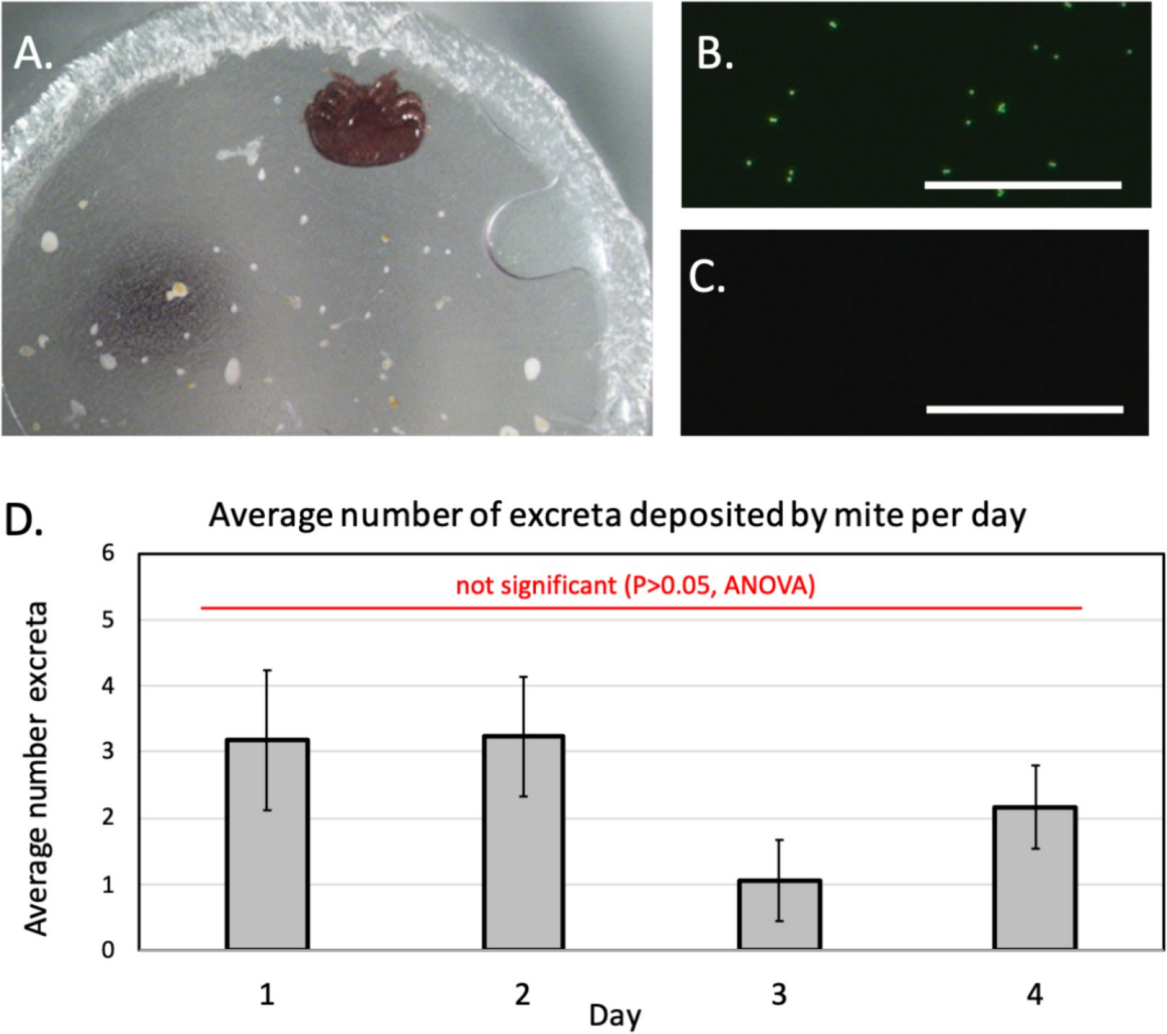
Evidence supporting *Varroa* mites fed on the artificial diet. (A) Photograph shows the parafilm membrane with a live mite attached and numerous excretory deposits. (B) Micrograph showing fluorescent microbeads in mite feces collected from devices housing mites provided diet containing microbeads. (C) Micrograph showing no fluorescent microbeads in mite feces collected from devices housing mites provided diet containing no microbeads. (D) The mean (± SE) total daily number of excretions deposited by mites on the membrane sachet. Measurement bar = 50 μM.

Evidence for feeding was supported by observing numerous excretory deposits (Fig 4A, S3 Table) from mites fed on honey bee pupae or the artificial diet; only minute amounts of excreta was observed from mites provided only water, indicating a lack of feeding. Evidence for feeding also came from finding FITC-labeled fluorescent beads in excreta of the mites fed on the diet containing these fluorescent microbeads (Fig 4B), but not in excreta from mites fed on the diet lacking the beads (Fig 4C). The daily average deposition of excreta was 2.55 ± 0.52 deposits per mite feeding on the artificial diet. Statistical analysis (one-way ANOVA) of the excreta data for each of the four days showed no significant differences in excreta per live mite per day (df = 35, *P* = 0.379, Fig 4D).

### *Varroa*-mediated vectoring and transmission of viruses to honey bee pupae

**Fig 5:**
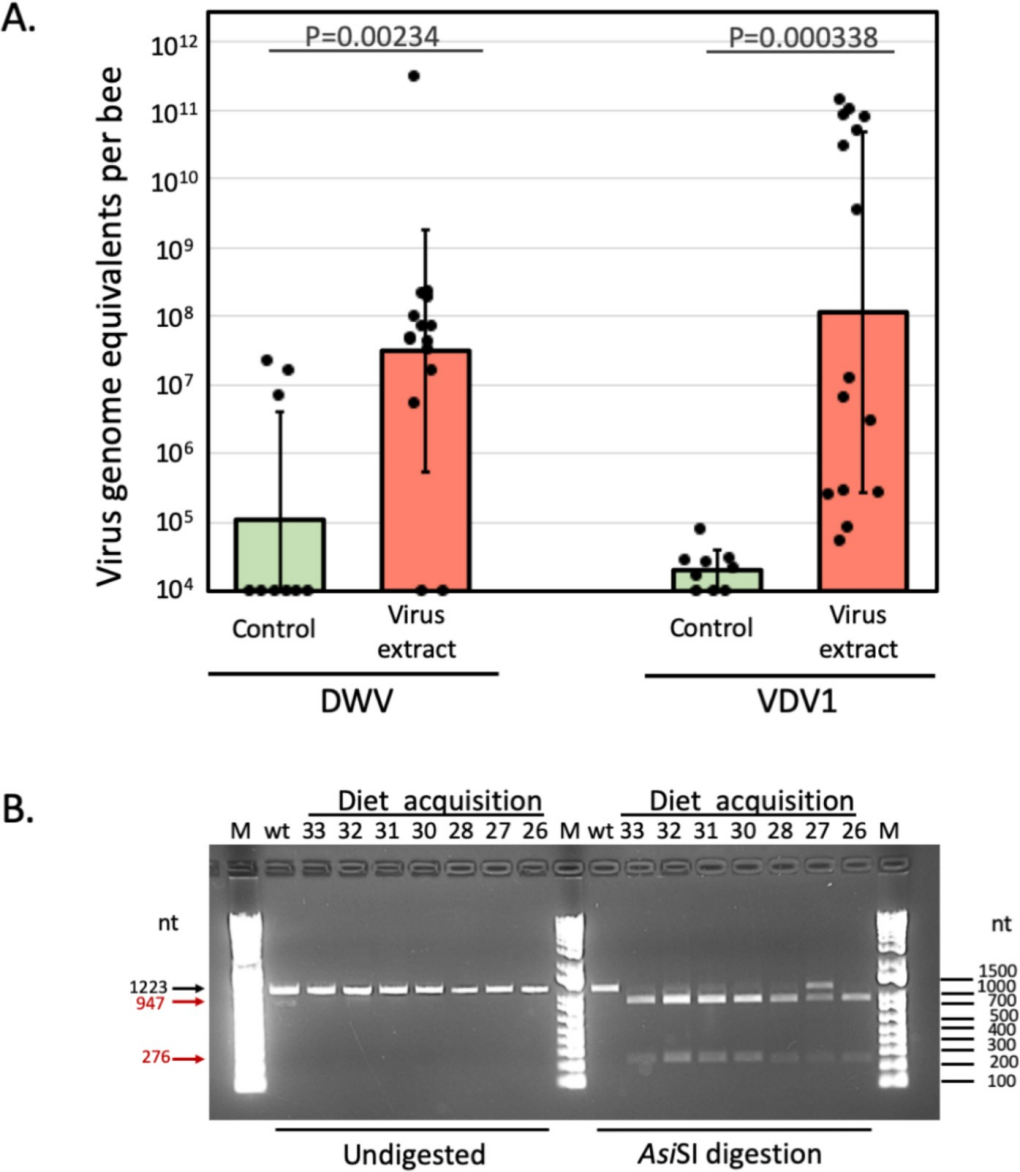
Transmission of VDV-1 and DWV acquired by *Varroa* mites from artificial diet to honey bee pupa. (A) Levels of clone-derived VDV-1 and wild-type DWV in honey bee pupae exposed to the *Varroa* mites which acquired the virus from the diet. Black dots indicate virus load in individual honey bees. The columns show the average virus genome equivalent per μL numbers for each treatment ±one standard deviation. Statistically significant differences from *post-hoc* unpaired Student’s t-test are indicated with *P*-values above the bars. Numerical values underlying the summary graphs are provided in S6 Table. (B) To confirm the VDV-1 clonal identity, the 1250 nt RT-PCR fragments corresponding to the 5’-terminal region of DWV and VDV-1 RNA genomes were amplified with the primers specific to both DWV and VDV-1 using RNA extracts from the experimental pupae with the virus levels exceeding 10^9^ genome copies from the “Virus extract” group (S6 Table), and from the wild-type VDV-1 and DWV-infected pupae, “wt”. The undigested 1250 nt fragments (left) and *Asi*SI-digested (right). Expected fragment sizes, undigested (black arrow) and *Asi*SI-digested (red arrows), are shown on the left, DNA ladder sizes are shown on the right.

The levels of honey bee actin mRNA detected from the RT-qPCR analyses were similar between pupae from the treatment and control groups (One-way ANOVA, *P* = 0.1395, S6 Table). One-way ANOVA of the virus accumulation in the control and treatment groups were highly significant, (*P*<0.01) for DWV (*F* = 11.83, df = 23 *P* = 0.00234; Cohen’s *d* = 1.473107, large effect size), and (*P*<0.001) for VDV-1 (*F* = 17.95, df = 23. *P* = 0.00034, Cohen’s *d* = 2.009988, large effect size) (Fig 5A). Importantly, high levels of DWV and VDV-1 exceeding 10^9^ virus copies per pupa were observed only in the treatment group. High levels of VDV-1 were observed in 7 of 15 pupae of the treatment group pupae, while only one pupa developed high DWV levels in the control group (Fig 5A). This result could be explained by a 1000-fold difference between the levels of DWV and VDV-1 in the diets containing 3.2 × 10^4^ and 3.3 × 10^7^ genome equivalent per μL respectively. Notably, 6 out of 15 pupae in the treatment group pupae containing more than 10^10^ genome equivalents of VDV-1 and reaching 1.38 × 10^11^ (Fig 5B, S6 Table). The amount of VDV-1 in a single pupa, therefore, was 388 times higher than the total amount of VDV-1 (3.3 × 10^8^ genome equivalents) contained in the 10 μL of artificial diet. The engineered viruses did not have any significant effect on mite survival (S1 Fig, S4 and S5 Tables).

We confirmed that VDV-1 derived from the artificial diet replicated in the treatment group pupae to high levels based on the diagnostic *Asi*SI restriction site (Fig 5B). This restriction site was not present in the wild-type isolates of VDV-1 or DWV (Fig 5B, lane “wt”). Further evidence showing that the VDV-1 transmitted to pupae was the cDNA clone VDV-1 acquired from the artificial diet that came from digesting the RT-PCR fragment corresponding to the 5’ terminal 1200 nt with *Asi*SI restriction enzyme. All seven of the recipient pupae that developed high virus levels showed that the *Asi*SI site was present in VDV-1 (Fig 5B). Notably, the sample isolated from the pupa 27 of the treatment group contained high levels of both DWV and VDV-1 (S5 Table), but only clone-derived VDV-1 was digested (Fig 5B, lane 27) which could be explained by the presence of wild-type DWV and wild-type VDV1, genomes of which did not have *Asi*SI restriction site.

## Discussion

Here we report on *Varroa*-mediated transmission of DWV-like viruses to honey bee pupae by employing a simple *in vitro* system that maintained *Varroa* mites on an artificial diet free of honey bee-derived components. We demonstrated that DWV and VDV-1 virus particles remained viable in an artificial diet medium, and that the viruses remained infectious in mite hosts and could be transmitted to honey bee host pupae. Notably, results showed that it could take less than 36 h for *Varroa* mites to acquire sufficient numbers of viral particles from feeding to transmit them to a honey bee host pupa, wherein they proliferated to high levels, *e*.*g*., 10^11^ genome equivalents per bee (see, ‘Virus extracts’ in Fig 5A). In contrast, no viral transmission to host pupae occurred subsequent to mites feeding on the ‘Control diet’ supplemented only with fluorescent microbeads.

A significant challenge for studying viral infection of honey bees is the tracking of specific viral strains during *Varroa*-mediated vectoring and transmission, while also accounting for the ‘background’ levels of DWV-like viruses present in both the recipient honey bee pupae and the *Varroa* mites. This challenge was resolved by using cDNA clone-derived VDV-1 (also known as DWV-B) that was tagged with a rare *Asi*SI restriction enzyme site, which allowed the dynamics of this specific viral strain to be accurately tracked, and to differentiate it from the ‘background’ viruses. For example, the significantly higher levels of wild-type DWV (DWV-A) detected in pupae from the ‘Virus extract’ group and in the virus extract itself can be accounted for by the additional wild-type DWV present in the pupae which was used to generate the VDV-1 particles by injecting VDV-1 *in vitro* transcript. Indeed, the pupae exposed for 36 h to mites previously fed on the diet lacking the virus had undetectable or low levels of DWV and VDV-1, below 10^8^ and 10^5^ genome equivalents, respectively (Fig 5A), although these mites were collected from the colonies harboring DWV. This latter finding supports the notion that DWV does not propagate nor persist in the *Varroa* mite vectors [14].

Although the longevity of *Varroa* mites using our system was often fewer than 5 days, which is similar to data reported elsewhere [19, 20], it was sufficiently long for our experiments. In contrast to the previous studies of *in vitro* systems for maintaining *Varroa* mites that did not confirm mite feeding [18–20, 22], in our study, the accumulation of mite excreta and the presence of the fluorescent microbeads acquired from the diet in mite excreta confirmed that mites had accessed, and indeed fed on the artificial diet. Notably, the greatest number of excretory deposits containing microbeads can be observed in the devices wherein mites had, presumably, fed the most, and survived the longest (S3 Table). Thus, the important question was not whether mites fed on the artificial diet survived as long as those fed on pupae [29], but whether mites survived to acquire, vector and transmit the viruses.

The experiment of *Varroa*-mediated vectoring and transmission of DWV-like viruses to honey bee pupae presented in this report validates the utility of the *in vitro* system for *Varroa* mite research. We demonstrated that mites could acquire viruses from an artificial diet free of honey bee cells and cellular components, then transmit virus particles to naïve hosts. Including such an artificial diet is particularly important in the light of the recent finding that *Varroa* mites can ingest honey bee cells (including fat body cells) rather than just hemolymph [14, 31]. Feeding mites on a diet free from honey bee cells will provide definitive insights into which mite-vectored viruses are capable of replicating and/or persisting in their mite carriers without the possible interference from viruses present in ingested cells. The *in vitro* system described in this report may be used for studies of *Varroa*-mediated vectoring and transmission of other honey bee viruses [17, 32]. However, the limitations of the currently known artificial diets is a confounding obstacle to other applications of the device where sustained mite survival and reproduction is needed. Further improvements to the nutritional value of these artificial diets are warranted, so as to make it possible to maintain *Varroa* mites *in vitro* for prolonged periods and to promote their reproduction.

## Supporting information

S1 Fig Mite survival analysis of the virus acquisition and transmission experiment. (PDF)

S1 Table Primers and the synthetic gene used in this study. (XLSX)

S2 Table Mite survival. (XLSX)

S3 Table Mite excreta data. (XLSX)

S4 Table Virus acquisition and transmission experiment: Mite survival summary. (XLSX)

S5 Table Virus acquisition and transmission experiment: Mite survival analysis. (XLSX)

S6 Table Virus acquisition and transmission experiment, RT-qPCR quantification of VDV-1, DWV and honey bee actin. (XLSX)

S1 Text Design of the infectious cDNA clone of *Varroa destructor* virus-1 (VDV-1) and production of the clone-derived VDV-1 inoculum. (PDF)

S2 Text Nucleotide sequence of the *Varroa destructor* virus-1 infectious cDNA construct (GenBank accession number MN249174). (PDF)

S1 Video *Varroa* mite feeding on artificial diet supplemented with the particles of honey bee viruses. (TXT)
