# Supplementary figures and images for: Varroa destructor mites vector and transmit pathogenic honey bee viruses acquired from an artificial diet
Source: PLoS One. 2020 Nov 24;15(11):e0242688. doi: 10.1371/journal.pone.0242688 (PMC7685439; doi:10.1371/journal.pone.0242688)

# Varroa survival

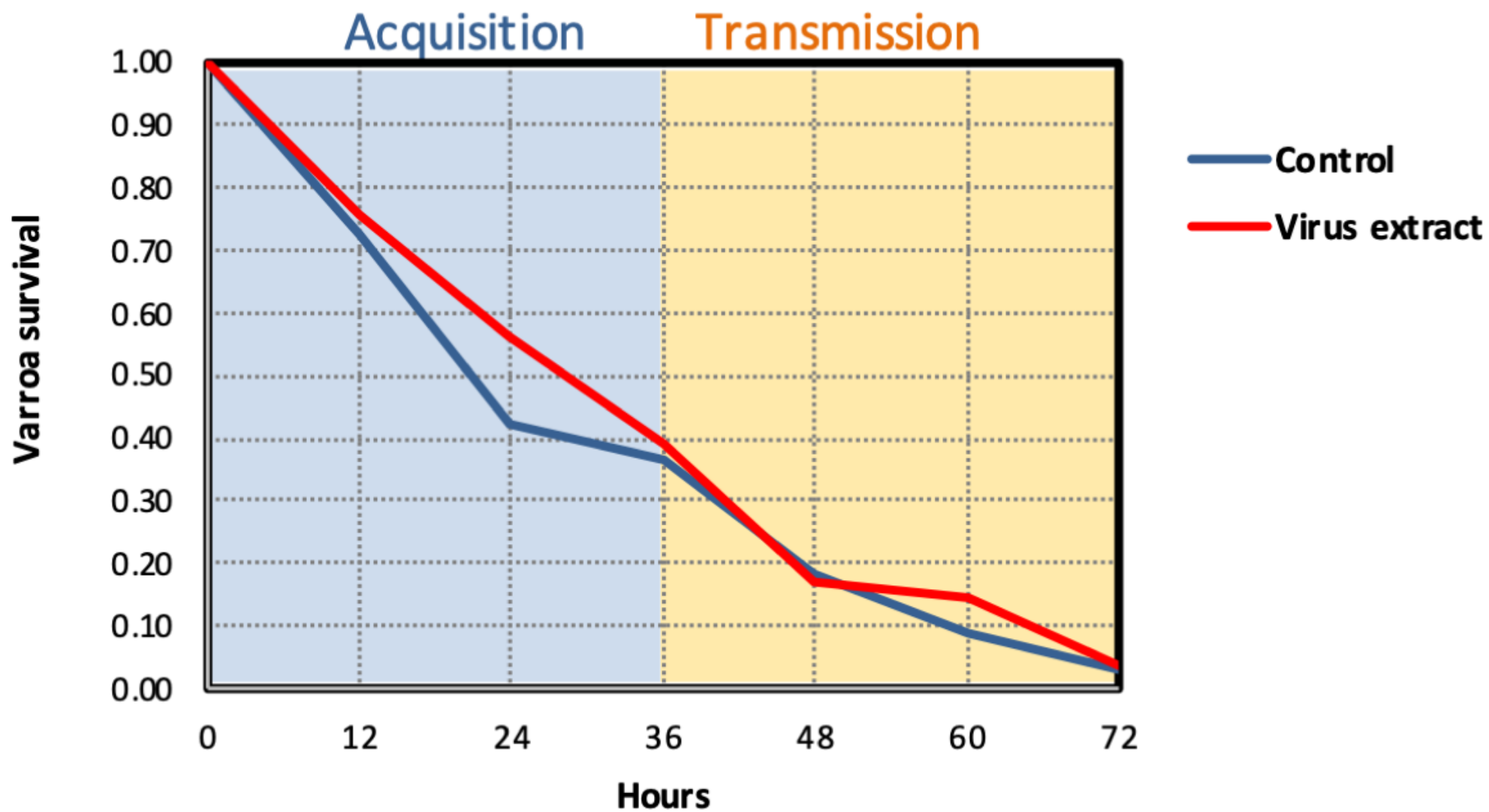

Wilcoxon test:  $P=0.5518$  (non-significant), Chi Square = 0.3541, DF = 1.

Supplement: S1 Fig — (PDF) [file pone.0242688.s001.pdf]
