# Supplementary material for: Varroa destructor mites vector and transmit pathogenic honey bee viruses acquired from an artificial diet
Source: PLoS One. 2020 Nov 24;15(11):e0242688. doi: 10.1371/journal.pone.0242688 (PMC7685439; doi:10.1371/journal.pone.0242688)
Supplement: S1 Text — (PDF) [file pone.0242688.s008.pdf]

S1 Text. Design of the infectious cDNA clone of Varroa destructor virus-1 (VDV1) and production of the clone-derived VDV1 inoculum

The full-length cDNA clone of Varroa destructor virus (VDV1) GenBank Accession number MN249174, was generated using total RNA from the honey bees sourced in California in 2016, isolate CA-07-2016, which showed high VDV1 and low DWV levels [1], using approach described in [2]. Two overlapping VDV1 cDNA fragments corresponding to the 5' and 3' sections of genomic RNA were amplified by RT-PCR using Superscript III reverse transcriptase (Invitrogen) and proof-reading Phusion DNA polymerase (New England Biolabs) to minimize amplification errors. The 5' VDV1 cDNA fragment, positions 277-6333 nt, was generated using “Pr-VDV1-6300R” as a reverse transcription primer and “VDV1NotFseAsiSi-F” and “Pr-VDV1-6300R” as PCR primers (S1 Table). The 3' VDV1 cDNA fragment, positions 4925-10148, was generated using “VDV1-AsciPmeI-A27R” as a reverse transcription primer and “Pr-VDV1-4800F” and “VDV1-AsciPmeI-A27R” as PCR primers, the primer sequences are given in Supplementary Table 1. These RT-PCR fragments containing overlapping 5' and 3' parts of VDV1 cDNA were cloned into the plasmid vector pTOPO-XL vector (Invitrogen) according to the manufacturer's instructions to produce constructs pVDV1-12 and pVDV1-3, correspondingly. The cloned cDNA inserts were Sanger-sequenced to confirm integrity of the protein coding sequences and homology with the previously sequenced VDV1 isolates. The *Not*I-*Hind*III 4.9 kb fragment of pVDV1-12 was inserted into the *Not*I-*Hind*III-digested pVDV1-3 to produce a construct containing nearly full-length VDV1 cDNA clone, positions 277-10148 nt. Unique restriction sites *Not*I and *AsiS*I, which were introduced at the 5' end of the cloned cDNA, were used to insert synthetic DNA sequence “T7-Rib-VDV1-5end” corresponding to the first 284 nucleotides of VDV1 preceded by the T7 RNA polymerase promoter and the ribozyme sequences [3] The resulting plasmid construct pVDV1-4, which contained introduced restriction site *AsiS*I in the IRES sequence at the position 277 nt, was linearized with *Pme*I restriction enzyme at the site located downstream the 3' terminal polyA sequence to generate the template for *in vitro* transcription. The *in vitro* transcription was carried out with T7 RNA polymerase (HighScript, New England Biolabs) for 3 hours at +37°C, the template plasmid DNA

was removed by digestion with RNAse-free Turbo DNAse (Ambion), the full-length VDV-1 transcripts were purified by RNeasy column (Qiagen).

To produce clone-derived VDV1 inoculum, 5 μg of the purified *in vitro* RNA transcript in 10 μL of phosphate-buffered saline (PBS), was injected into the hemolymph of purple eye honeybee pupae using syringes with a 0.3 mm 31G hypodermal needle G31 (BD Micro-Fine). The injected pupae were incubated 4 days at +33°C to allow development of the clone-derived virus infection and then were used to prepare tissue extracts containing the clone-derived VDV1 virus particles. The extracts were produced by homogenizing individual pupa with 1 mL of PBS, subjecting the homogenate to three cycles of freezing and thawing, clarifying extract by low-speed centrifugation (3000 rpm for 5 minutes), and filtering through a 0.22 μm nylon syringe filter. Concentrations of VDV1 and DWV was determined by qRT-PCR and identity of the clone-derived VDV1 was confirmed by sequencing and restriction digestion of the VDV1 IRES region.

References:

1. Ryabov, E.V., Childers, A.K., Chen, Y., Madella, S,. Nessa, A., vanEngelsdorp, D., Evans, J. D. *(*2017) Recent spread of Varroa destructor virus-1, a honey bee pathogen, in the United States. *Scientific Reports* 7: 17447. https:// doi.org/10.1038/s41598-017-17802-3 PMID: 29234127
2. Ryabov E.V., Childers, A.K., Lopez, D., Grubbs, K., Posada-Florez F., Weaver, D., Girten, W., vanEngelsdorp, D,. Chen, Y., Evans, J.D. (2019) Dynamic evolution in the key honey bee pathogen deformed wing virus: Novel insights into virulence and competition using reverse genetics. *PLoS Biology* 17:e3000502. doi: 10.1371/journal.pbio.3000502
3. Herold, J,. Andino, R. (2000) Poliovirus requires a precise 50 end for efficient positive-strand RNA synthesis. *J. Virology* 74:6394–400. https://doi.org/10.1128/jvi.74.14.6394-6400.2000 PMID: 10864650
