# Supplementary material for: Varroa destructor mites vector and transmit pathogenic honey bee viruses acquired from an artificial diet
Source: PLoS One. 2020 Nov 24;15(11):e0242688. doi: 10.1371/journal.pone.0242688 (PMC7685439; doi:10.1371/journal.pone.0242688)
Supplement: S2 Text — (PDF) [file pone.0242688.s009.pdf]

S2 Text. Nucleotide sequence of the Varroa destructor virus-1 infectious cDNA construct (GenBank accession number MN249174).

```
LOCUS       Synthetic              10272 bp    mRNA    linear   VRL 29-JUL-2019
DEFINITION  construct Varroa destructor virus 1-California-2016 infectious cDNA
            clone 4.
ACCESSION   Synthetic
VERSION
KEYWORDS    .
SOURCE      Varroa destructor virus 1
  ORGANISM  Varroa destructor virus 1
            Viruses; Riboviria; Picornavirales; Iflaviridae; Iflavirus.
REFERENCE   1  (bases 1 to 10272)
  AUTHORS   Ryabov,E.V.
  TITLE     Full-length cDNA infectious clone of Varroa destructor virus-1, a
            pathogen of the honeybee, Apis mellifera
  JOURNAL   unpublished
REFERENCE   2  (bases 1 to 10272)
  AUTHORS   Ryabov,E.V.
  TITLE     Direct Submission
  JOURNAL   Submitted (29-JUL-2019) USDA-ARS Bee Research Laboratory, United
            States Department of Agriculture, Beltsville Agricultural Research
            Center, 10300 Baltimore Avenue, Bldg. 306, Beltsville, MD 20705,
            USA
COMMENT     Bankit Comment: Vecscreen Comment:Submitter says that this sequence
            is a Synthetic Construct
            Bankit Comment: ALT EMAIL:eugene.ryabov@ars.usda.gov
            Bankit Comment: TOTAL # OF SEQS:1

            ##Assembly-Data-START##
            Sequencing Technology :: Sanger dideoxy sequencing
            ##Assembly-Data-END##
FEATURES             Location/Qualifiers
     source          1..10272
                     /organism="Varroa destructor virus 1"
                     /mol_type="mRNA"
                     /cultivar="California CA-07-2016 (APHIS ID 2806"
                     /isolate="California CA-07-2016 (APHIS ID 2806)"
                     /host="Apis mellifera"
                     /db_xref="taxon:232800"
                     /clone="pVDV1-California-2016-No-4"
                     /country="USA"
                     /collection_date="2016"
     gene            89..10264
                     /gene="Varroa destructor virus 1"
                     /gene_synonym="VDV1 cDNA"
                     /note="full-length cDNA copy of VDV1 genomic RNA"
                     /nomenclature="Varroa destructor virus 1"
     CDS             1242..9923
                     /gene="Varroa destructor virus 1"
                     /gene_synonym="VDV1 cDNA"
                     /note="leader protein, structural proteins, non-structural
                     proteins: helicase, protease, RNA-dependent RNA polymerase
                     domains; [intronless gene]"
                     /codon_start=1
                     /product="Viral polyprotein"
                     /translation="MAFSCGTLSYAAVAQAPSVAHAPRSWEIDEARRRRVIKRLALEQ
                     ERIRNVLDVTVYDHTTWEQEDARDNEFLTEQLNNLYTIYSIAERCTRRPVQEHVPISI
                     SNRYSPLESLKIEVGKDAGEFVFKKPKYTKICKKVKRVTSKFVREKVVRPVCNRSPML
                     LFKIKKVIYDLHLYRLRKQVRLLRREKQREYELECVTSLLQLSNPVSAKPEMDNPNPG
                     PDGEGEVELEKDSNVVLTTQRDPSTSIPAPTSVKWSRWTSNDVVDDYATITSRWYQIA
                     EFVWSKDDPFDKELARLILPRALLSSIEANSDAICDVPNTIPFKVHAYWRGDMEVRVQ
                     INSNKFQVGQLQATWYYSDHENLNIQTKRSVYGFSHMDHALISASASNEAKLVIPFKH
                     VYPFLPTRVVPDWTTGILDMGTLNIRVIAPLRMSATGPTTCNVVVFIKLNNSEFTGTS
                     SGKFYANQIRAKPEMDRVLNLAEGLLNNTVGGCNMDNPSYQQSPRHFVPTGMHSLALG
                     TNLVEPLHALRLDASGTTQHPVGCAPDEDMTVSSIASRYGLIRQVQWKKDHAKGSLLL
                     QLDADPFVEQKIEGTNPISLYWFAPVGVVSSMFMQWRGSLEYRFDIIASQFHTGRLIV
                     GYVPGLTASLQRQMDYMKLKSSSYVVFDLQESNSFTFEVPYVSYRPWWVRKYGGNYLP
                     SSTDAPSTLFMYVQVPLIPMEAVSDTIDINVYVRGGSSFEVCVPVQPSLGLNWNTDFI
                     LRNDEEYRAKNGYAPYYGGVWHSFNNSNSLVFRWGSASDQIAQWPTITVPRGELAFLR
                     IRDAKQAAVGTQPWRTMVVWPSGHGYNIGIPTYNAERARQLAQHLYGGGSLTDEKAKQ
                     LFVPANQQGPGKVSNGNPVWEVMRAPLATQQAHIQDFEFVEAVPEGEESRNTTVLDTT
                     TTLQSSGFGRAFFGEAFNDLKTLMRRYQLYGQLLLSVTTDKDIDHCMFTFPCLPQGLA
                     LDIGSAGSPHEIFNRCRDGIIPLIASGYRFYRGDLRFKIVFPSNVNSNIWVQHRPDRR
```

LKGWSEAKIVNCDAVSTGQGVYNHGYASHIQITRVNNVIELEVPFYNATCYNYLQAFN
PSSAASSYAVSLGEISVGFQATSDDIAAIVNKPVTIYYSIGDGMQFSQWVGYQPMMIL
DQLPAPVVRAVPEGPIAKIKNFFHQTADEVREAQAAKMREDMGIVVQDVIGELSQAIP
DLQQPEVQANVFSLVSQLVHAIIGTSLKTVAWAIVSIFVTLGLIGREMMHSVITVVKR
LLEKYHLATQPQESANSGTVISAIPEAPNAEAEEASAWVSIIYNGVCNMLNVAAQKPK
QFKDWVKLATVDFSNNCRGSNQVFVFFKNTFEVLKKMWGYVFCQSNPAARLLKAVNDE
PEILKAWVKECLYLDDPKFRMRRAHDQEYIERVFAAHSYGQILLHDLTAEMNQSRNLS
VFTRVYDQISKLKTDLMEMGSNPYIRRECFTICMCGASGIGKSYLTDSLCSELLRASR
TPVTTGIKCVVNPLSDYWDQCDFQPVLCVDDMWSVETSTTLDKQLNMLFQVHSPIVLS
PPKADLEGKKMRYNPEIFIYNTNKPFPRFDRIAMEAIYRRRNVLIECKANEEKKRGCK
HCENNIPIAECSPKILKDFHHIKFRYAHDVCNSETTWSEWMSYNEFLEWITPVYMANR
RKANESFKMRVDEMQMLRMDEPLEGDNILNKYVEVNQRLVEEMKAFKERTLWADLQRV
GSEISTSVKKALPTISITERLPHWTIQCGIAKPEMDHAYEVMSSYAAGMNAEIEAHEQ
VRRSSLECQYIEPSTSRPLDEEGPTIDEELLGEVEFTSSALERLVDEGYITGKQKKYM
ATWCTKRREHVSDFDLVWTDNLRVLSAYVHERSTSTRLSTDDVKLFKTISMLHQRYDT
TDCAKCQHWYAPLTAIYVDDRKLFWCQKETKTLIDVRKLSKEDVTVQSKLINLSVPCG
DVCMLHSKYFNYLFHKAWLFENPTWRLIYNGTKKGMPEYFMNCVDEISLDSKFCKVKV
WLQAIIDKYLTRPVKMIRDFLFKWWPQVAYVLSLLGIIGITAYEMRNPKSTAEDLAEH
YVNRHCNSDFWSPGMATPQGLKYSEAITAKAPRIHRLPVSTRPQGSTQQVDAVVNKIL
QNMVYIGVVFPKGPGSKWRDINFRCLMLHNRQCLMLRHYIESTAAFPEGTKYYFKYIH
NQETRMSGDISGIEIDLLSLPRLYYGGLAGEESFDSNIVLVTMPNRIPECKSIVKFIA
SHAEHARAQNDGVLVTGEHTQLLAFENNNKTPISINADGLYEVILQGVYTYPYHGDGV
CGSILLSRNLQRPIIGIHVAGTEGLHGFGVAEPLVHEMFTGKAIESEREPYDRVYELP
LRELDESDIGLDTDLYPIGRVDAKLAHAQSPSTGIKKTLIHGTFDVRTEPNPMSSRDP
RIAPHDPLKLGCEKHGMPCSPFNRKHLELATTHLKEKLISVVKPINGCKIRSLQDAVC
GVPGLDGFDSISWNTSAGFPLSSLKPPGSSGKRWLFDIELQDSGCYLLRGMRPELEIQ
LTTTQLMRKKGIKPHTIFTDCLKDTCLPVEKCRIPGKTRIFSISPVQFMIPFRQYYLD
FMASYRAARLNAEHGIGIDVNSLEWTNLATSLSKYGTHIVTGDYKNFGPGLDSDVAAS
AFEIIIDWVLNYTEEDDKDEMKRVMWTMAQEILAPSHLCRDLVYRVPCGIPSGSPITD
ILNTISNCLLIRLAWQGITDLPLSEFSRHVVLVCYGDDLIMNVSDEMIDKFNAVTIGD
FFSRYKMEFTDQDKSGNTVRWRTLQTATFLKHGFLKHPTRPVFLANLDKVSIEGTTNW
THARGLGRRVATIENAKQALELAFGWGPEYFNHVRNTIKMAFDKLGIYEDLITWEEMD
VRCYASA"

BASE COUNT   3022 a   1647 c   2320 g   3283 t

ORIGIN

1 gcggccgcgg ccggccgcta taatacgact cactataggg attttaaact gatgaggccg
61 aaaggccgaa aacccggtat cccgggttct ttaaaattcg ctatgggagg cgatttatgc
121 cttccatagc gaattacggt gcaactaaca attttagata gtagccatga acaaacatta
181 tgattactca ctacgtattg atcattttta caatgacttg cgtagcatga agcgcatgct
241 tgtagttata actatgttat tttgcaagtt ggagataatt gtattggatt atggatgcgt
301 gcactaagtg tctacatcta tagtcgtttg tggttcaagt tttgtgtta gtagtacaat
361 tttgagcgat cgcagtatcg tatgaatgat atttgaatga caacactgaa gtataaaata
421 tataaaatcc aaaaatattt ttaatcttat tcagtgtagt gtttgataga gtagaatgcc
481 atgtgaccgc tcaaagaagt ccattatggt atatcattcg aagtcgaata cttgtgtata
541 gttattgtat tttattagta atattagtag tccgtaacta tcataatcct attatagttt
601 gattatatga tagaccactg cagtatcgag tagagtttag aaagagtagt gcaatagtaa
661 gatcactgtc accgaccact cattgtaata gtgaggttcg tcggaaacca gttattgtgc
721 agcgactagc aatcgtgaat caatatagtt ggtattctaa atatgagacg attcggcgat
781 tttattgcga ctgaaatttc atatttagca tgtcaggtct tattatgaat gctcgagtat
841 ttatttctgc ggtagagtag ggacccctct atctctcagg tactgtatga ggcgaaagtg
901 tgaaagtaat ttatgtctct atacataagt gactgtattg ggatttcctt tggcaagaat
961 cctttcaata cagtataatt tatgccacgg tacgttacgt tcgcagggca cccgttaatg
1021 tcacatagcc cagacgatga cgaatggaaa gacattactt tttattttaa tgctacgatt
1081 attgctgttt tattttgctg ttttaatttg ctattatatt ttgctatttt cattattgct
1141 aaatatattt ctttgctatt tttgctttat atattagatt caattctttt tattttatat
1201 tttcaatttg attttgagtt tgaaggtaaa tatatataaa aatggcattt agttgtggaa
1261 ctctttctta tgctgctgtt gcccaagctc cctctgtagc tcatgctccc cgtagttggg
1321 agattgatga agctaggcgt cgacgcgtta ttaagcgttt ggcgttggaa caggaacgga
1381 ttcgaaatgt tcttgacgtc actgtgtatg atcatacaac gtgggagcaa gaggatgcgc
1441 gtgacaatga gttccttacg gaacaattga ataatttata tacgatatat tctatagctg
1501 aaagatgtac ccgccgccct gttcaagaac atgtccccat ttcaatcagt aatagatatt
1561 ccccttttaga atcccttaag attgaggtag gaaaagacgc aggtgagttc gtatttaaga
1621 aacccaaata tacaaagatt tgtaagaaag tgaaacgggt gacatcaaaa tttgtgcgcg
1681 agaaagttgt taggcccgtt tgtaatcgat cgcccatgtt attatttaaa attaagaaag
1741 taatatatga tttacatttg tatcggttac ggaaacaagt tcggcttctc agacgcgaaa
1801 aacagcgtga atatgagtta gagtgtgtta ctagtttgct acagttatct aatcctgttt
1861 cagctaaacc tgagatggac aatcctaatc ctggtccaga tggtgaaggt gaagttgaat
1921 tagaaaagga tagtaatgta gtattaacta cacaacgtga tcctagtacc tctattcctg
1981 ctccaactag tgtgaagtgg agtagatgga ccagtaatga tgttgtggat gattatgcca
2041 ctataacttc gcgttggtat caaattgccg aatttgtatg gtcaaaggat gatccatttg
2101 ataaggaatt ggcgcgctta attttacctc gagctttgtt atctagtatt gaggctaatt
2161 ctgacgctat ttgtgatgta cctaatacta ttccgtttaa ggtacatgca tattggcgtg
2221 gagatatgga agttcgagtg cagattaact cgaataaatt ccaggttggt caattacagg
2281 caacttggta ctattcggat catgaaaatt tgaatatcca gacgaagcga agtgtgtatg
2341 gtttttcgca tatggatcat gctctgatta gcgcatcagc gagtaatgaa gcaaaattag
2401 tgatacctt taaacacgta tatccattct taccaacgcg tgtcgttcct gattggacaa
2461 ctggtattct tgatatgggt accttaaata ttcgtgtaat tgctccacta cgtatgagtg

2521 cgacgggacc aaccacttgt aatgttgtag tatttattaa gttaaataat agtgaattta
2581 ctggtacttc ttctggtaag ttttacgcga atcaaattag ggcaaaacct gaaatggacc
2641 gtgtgctaaa tttggcagaa ggattactaa ataatactgt aggtggttgt aatatggata
2701 atccgtcata tcagcaatct ccgcgtcatt ttgttcctac tggtatgcat agtttagctt
2761 taggcactaa tttagtagag cctttgcatg cattacgatt agatgcatca ggtacaacac
2821 aacatccagt tgggtgtgcg cctgatgaag atatgactgt atcttccatt gcatcacgat
2881 atggtttaat tcgccaagtg caatggaaga aagaccatgc gaaaggatca ttattattac
2941 aacttgatgc tgatcctttc gttgaacaga aaattgaggg aaccaatcca atttctttgt
3001 attggtttgc tccggttgga gtcgtatcta gtatgttcat gcaatggaga ggttctttag
3061 aatatagatt tgatattata gcttcccaat ttcatacggg taggttaatt gtaggttatg
3121 ttcctggact gactgcttct ttacaacgtc aaatggacta tatgaaattg aagtcatcta
3181 gttatgtggt gtttgattta caggaaagta atagttttac gtttgaagtg ccctatgtgt
3241 catacagacc gtggtgggtg cgtaagtatg gtggtaatta tctgccatct tctactgatg
3301 cgcctagcac actgtttatg tatgtacaag taccattgat acctatggaa gctgtttctg
3361 atactataga tatcaatgtg tatgtgcgtg gtggcagttc gtttgaggtt tgtgttccag
3421 tccaacctag tttaggtttg aactggaata cagatttcat attacgtaat gatgaggagt
3481 accgcgcaaa gaatggatat gcaccatatt atggtggtgt gtggcatagc ttcaataata
3541 gtaattcgct tgtttttaga tggggttcgg cttcagatca aattgctcaa tggccaacaa
3601 taacagtgcc tcgaggagag ttagcattcc tgcgtatccg cgatgctaag caagctgctg
3661 taggaacgca accttggcgt actatggtcg tttggccttc aggtcatgga tataatattg
3721 gaataccaac ttataatgct gaacgagcaa gacaacttgc tcagcatttg tatggtggtg
3781 ggtctttgac agatgaaaaa gctaagcaat tatttgtgcc tgctaaccag caaggacccg
3841 gcaaagtaag taatggtaac cccgtctggg aagtaatgcg cgcgcctctt gcaactcagc
3901 aagcgcatat acaagatttt gaatttgttg aagctgttcc agaaggcgaa gaatcacgca
3961 acactacggt gctagacacg acaacaacgt tacagtctag cggatttggt cgcgctttct
4021 tcggtgaggc atttaacgat cttaagacgt taatgcgccg ataccaatta tatggtcaat
4081 tattgttatc cgttactacg gataaggata ttgatcattg tatgtttacc ttcccttgtt
4141 tacctcaagg gttagcgtta gatataggtt cggctggatc gcctcatgaa atatttaatc
4201 gctgccgtga tggtatcatt ccgttgatag cgtcagggta tcggttttat cgaggcgatt
4261 tacggttcaa aattgttttc ccaagtaacg ttaatagcaa tatttgggta caacatcgac
4321 cagatcgtag attgaaagga tggtctgaag cgaaaatagt aaactgtgat gctgtatcta
4381 ctggacaagg tgtttataat catggatatg ctagtcatat tcagattacg cgtgtaaata
4441 atgttataga attggaagtc ccgttttata acgctacgtg ctataattat ttgcaagcgt
4501 ttaacccatc tagtgcagcg tcgagttatg ccgtttcgct cggagagatt tcggttggtt
4561 ttcaagctac tagtgatgac attgcagcca tagttaataa acctgtaact atatattaca
4621 gtattggcga tggtatgcag ttttcgcagt gggttggtta tcaaccaatg atgattctag
4681 accaattgcc agcaccagta gttagggctg tgcctgaggg ccctatagcg aagataaaga
4741 actttttcca ccaaacggca gatgaagttc gagaagctca ggccgcaaag atgcgtgaag
4801 atatgggtat agtagtccaa gacgttatag gagagttaag tcaggctata cccgatcttc
4861 aacaaccgga agttcaagcg aatgtttttt ctctggtgtc acagttagtg catgctatca
4921 tcggtactag tcttaagaca gttgcttggg cgattgtttc gatttttgta actttaggtt
4981 tgattggacg tgaaatgatg cattcagtca taactgtagt taagcggtta ttagaaaaat
5041 atcacttggc gacgcaaccc caggaatccg ccaattcagg tacggttatt tccgctattc
5101 cagaagcacc caatgctgaa gcagaggagg ccagtgcctg ggtatccatt atttataatg
5161 gtgtgtgtaa tatgttgaat gtagccgctc aaaaaccgaa acaatttaaa gattgggtaa
5221 aattagctac cgtagatttt agtaataatt gtagaggtag taatcaggta tttgtgtttt
5281 tcaagaatac gtttgaagtg ttgaagaaaa tgtggggtta tgtgttttgt cagagtaatc
5341 ctgcagcgcg actcttgaaa gcagtgaatg acgaacctga gattttaaaa gcgtgggtta
5401 aagaatgtct gtatttagat gatcctaaat ttagaatgcg acgtgcgcat gatcaagagt
5461 atattgagag agtgtttgcg gcccattcgt atggacaaat tttattgcat gacttaacgg
5521 ctgaaatgaa tcaatcgcgt aatttaagtg tgtttacgag agtgtatgat caaatatcta
5581 aattgaagac ggatctcatg gaaatgggat caaacccata tatcaggcgt gaatgcttta
5641 cgatttgtat gtgtggtgca tctggaattg gtaagtctta tttaactgat tctttatgca
5701 gcgagctctt acgtgcgagt cgtactccag tgacaacggg cattaagtgt gtcgtgaacc
5761 ctttgtctga ttattgggat cagtgtgatt ttcagcccgt tttatgtgtt gatgacatgt
5821 ggagtgttga aacgtctact acgctcgata aacagttaaa tatgttattc caggttcatt
5881 caccaattgt actttcaccg cctaaagctg atttagaagg taagaaaatg cgttataatc
5941 ctgaaatatt catatataat acgaataaac cttttccgag gtttgatcgt atagctatgg
6001 aagctattta tcgacgtaga aacgttttaa ttgaatgtaa ggctaatgaa gagaagaagc
6061 gtggatgtaa acattgtgag aataatatac ccattgctga atgtagtcca aaaattttga
6121 aagattttca tcacattaaa tttcgttatg ctcatgatgt gtgtaattct gaaactacgt
6181 ggtctgagtg gatgtcgtat aatgaatttt tggaatggat tactcctgta tatatggcta
6241 atcgacgtaa agcaaatgaa tcgtttaaga tgcgtgttga tgaaatgcaa atgttgcgta
6301 tggatgagcc cttggaaggc gataatattt taaataagta tgttgaagtt aatcagcgct
6361 tagttgagga aatgaaagct tttaaagagc gaaccctttg ggctgattta caacgtgttg
6421 gctcagagat tagtacttca gttaagaaag cattaccaac tatttccatt actgagaggc
6481 taccacattg gactatccaa tgtggcatag ctaagcctga aatggatcat gcttatgaag
6541 ttatgagttc atatgcagca ggaatgaacg cagaaattga agcgcatgaa caagttcgtc
6601 gttcttcttt ggaatgtcag tatattgagc cttcaacttc aagacctctg gatgaagagg
6661 gtcctactat cgacgaggaa ttacttggcg aagtagaatt tacttcttca gctttggagc
6721 gtttggttga tgaggggtat attactggta aacaaaagaa gtatatggca acttggtgta
6781 cgaaacgaag agagcatgta tccgattttg atttagtatg gacggataat ctgcgtgttt
6841 tgagtgcgta tgtccacgag cgttctacat ctacgcgttt atctaccgat gatgttaaat
6901 tatttaagac gattagtatg ttacatcaga ggtatgatac cactgattgt gcaaaatgcc
6961 aacattggta tgcaccatta acagctattt atgttgatga tagaaagcta ttttggtgcc
7021 agaaggagac taagactttg atagatgttc gtaaattgtc gaaagaggat gttacagtcc
7081 aatcgaaatt aattaactta tcggttccgt gcggtgatgt gtgtatgtta cattctaaat

```
     7141 actttaatta tttattccat aaagcgtggt tgtttgaaaa tccaacatgg cgtttaatat
     7201 ataatggtac taagaaaggt atgcctgagt atttcatgaa ttgcgtggat gaaatttcat
     7261 tagattcaaa attttgtaaa gtaaaggttt ggcttcaagc aattattgat aaatatttga
     7321 ctcgtccagt gaaaatgatt cgtgactttt tatttaaatg gtggccgcaa gtagcatacg
     7381 tgttaagttt gttaggtata attggtataa ctgcgtatga aatgcgtaat cctaaatcaa
     7441 cagcagaaga cttggctgag cactatgtta ataggcattg taattcagat tttggtcac
     7501 caggtatggc gacgcctcag ggattaaaat atagtgaagc gataacagct aaagcgccta
     7561 gaatccatag attgcccgtt agtactagac ctcagggatc aacgcagcaa gtggacgccg
     7621 ttgtgaataa gattttgcag aatatggtgt atatcggtgt tgtatttcca aaagggcctg
     7681 gtagtaagtg gcgagatatt aattttagat gtcttatgct tcataatcgg caatgtttga
     7741 tgttgcggca ttacattgag tcgacggctg cttttccgga gggtaccaaa tactatttta
     7801 agtatattca taatcaagaa actcgaatgt caggtgatat atctggtatt gagattgatt
     7861 tattgagttt acctagattg tattatggtg gcttagcggg ggaagagtcg tttgatagca
     7921 atatagtgtt agtaactatg ccgaatagaa ttcctgagtg taagagtatt gtgaagttta
     7981 tagcttcaca tgctgaacat gctcgtgctc aaaatgatgg tgtgttagtt actggtgaac
     8041 atactcagtt attggcgttc gagaataata ataaaacacc tataagtatt aatgctgatg
     8101 gtttgtatga ggttatactt caaggagtat acacttatcc ataccatggt gatggtgttt
     8161 gtgggtctat attattgtct cgtaatttac aacgaccgat tatagggatc catgtagctg
     8221 gtactgaagg attacatggc tttggtgttg ctgaacctct tgttcatgag atgttcactg
     8281 ggaaagcaat agagagtgaa agggaaccgt atgatcgtgt gtatgaatta cctttgcgtg
     8341 aattagatga atctgatata ggtttagata ctgacttata tcctatagga agagttgatg
     8401 cgaaattagc tcatgcccaa agtccttcaa caggaattaa aaagacgctt attcatggta
     8461 cttttgatgt tcggactgaa ccgaacccga tgtcatcacg agacccaaga atagcgccac
     8521 atgatccgtt gaagttaggg tgtgagaaac atggtatgcc atgttctcca tttaatcgaa
     8581 aacatttgga attagcaaca actcatttaa aggagaagtt aatttccgta gttaaaccta
     8641 taaacggatg caagattaga agtttgcagg atgctgtgtg tggtgtacca ggtttggatg
     8701 gctttgattc aatatcctgg aatactagtg ctggttttcc tttatcttca ttaaaaccgc
     8761 caggctcttc tggtaaacga tggttgtttg atattgaatt acaagattca ggatgttatc
     8821 ttttgagagg gatgagacct gaacttgaga tacagttgac aacaactcag ttaatgagga
     8881 agaagggaat aaagcctcac actatattca cggattgttt gaaagataca tgtttgcctg
     8941 tggaaaaatg cagaatacct ggtaagacta gaatatttag tataagtccc gtccaattta
     9001 tgattccatt tcgacaatac tatctcgatt ttatggcgtc gtaccgtgcc gctagactta
     9061 atgctgagca tggaataggt atagacgtga acagcttgga atggacaaac ttggcaacaa
     9121 gtctgtcgaa gtatggcacg catattgtga caggagatta caagaatttt ggtcctgggt
     9181 tagattctga tgttgccgct tcagctttcg aaattatcat tgattgggtg ttaaattaca
     9241 ctgaagaaga tgataaagac gaaatgaagc gtgtaatgtg gactatggct caggaaatct
     9301 tagctcctag tcacttatgt cgtgatttag tatatcgcgt accatgcggt attccttctg
     9361 gatcaccaat tacggacatt ttgaatacta tttcgaattg tttgttaatt cgattggctt
     9421 ggcaaggtat tactgatttg cctttatccg aattttctag acatgtcgtg ctagtttgtt
     9481 atggtgatga tctcatcatg aatgtaagtg atgagatgat agataaattc aacgctgtaa
     9541 caattggcga tttcttttcg cgatataaga tggaatttac ggatcaggat aaatctggaa
     9601 atacagtgcg gtggcgaact ttacaaactg ccacgttttt gaagcatggg ttcttgaaac
     9661 atccaacaag acccgtgttt ctagccaatc tggataaggt ttctatagaa ggaacaacca
     9721 attggacaca tgctcgagga ttgggtcgtc gagtagcaac cattgagaat gctaaacaag
     9781 cgctagagtt ggcattcgga tggggtcccg aatactttaa tcatgttcgg aataccatta
     9841 aaatggcatt cgacaagtta ggtatttatg aggatctcat cacatgggaa gaaatggatg
     9901 ttagatgtta tgctagcgcg taattttaag attttaatac tcattaaaat taatttgtat
     9961 ttaggttatt ggaattgagg gaagtaccac cccccaagac cttcgtttta aatctactaa
    10021 gaggagtgaa cttgcatata agagtctaaa agcagagtgg attagaccac cacttttagc
    10081 ttatatgtga ggaaggttga gttgcctcta aagactcagc tccgtagtag agtagtttta
    10141 gttacgatta aagtggtact ctaggttagg tgttactcgc gtattgtcgc ataacggcaa
    10201 tgcgtcctaa ttttagtata gttttaacca taatagtaaa aaaaaaaaaa aaaaaaaaaa
    10261 aaaagtttaa ac
//
```
